# Measuring the distribution of cellulose microfibril angles in primary cell walls by small angle X-ray scattering

**DOI:** 10.1186/1746-4811-10-25

**Published:** 2014-08-05

**Authors:** Friederike Saxe, Michaela Eder, Gunthard Benecke, Barbara Aichmayer, Peter Fratzl, Ingo Burgert, Markus Rüggeberg

**Affiliations:** 1 Department of Biomaterials, Max Planck Institute of Colloids and Interfaces, Potsdam-Golm, 14476, Germany; 2 Swiss Federal Institute of Technology Zurich, Institute for Building Materials, 8093 Zurich, Switzerland; 3 Swiss Federal Laboratories for Materials Science and Technology, Applied Wood Materials Laboratory, 8600 Duebendorf, Switzerland

**Keywords:** X-ray scattering, Primary cell wall, Cellulose microfibril orientation, *Chara corallina*, *Arabidopsis thaliana*

## Abstract

**Background:**

X-ray scattering is a well-established method for measuring cellulose microfibril angles in secondary cell walls. However, little data is available on the much thinner primary cell walls. Here, we show that microfibril orientation distributions can be determined by small angle X-ray scattering (SAXS) even in primary cell walls. The technique offers a number of advantages: samples can be analyzed in the native hydrated state without any preparation which minimizes the risk of artifacts and allows for fast data acquisition. The method provides data averaged over a specimen region, determined by the size of the used X-ray beam and, thus, yields the microfibril orientation distribution within this region.

**Results:**

Cellulose microfibril orientation distributions were obtained for single cells of the alga *Chara corallina,* as well as for the multicellular hypocotyl of *Arabidopsis thaliana*. In both, Chara and Arabidopsis, distributions with a broad scattering around mean microfibril angles of approximately 0° and 90° towards the longitudinal axis of the cells were found.

**Conclusions:**

With SAXS, the structure of primary cell walls can be analysed in their native state and new insights into the cellulose microfibril orientation of primary cell walls can be gained. The data shows that SAXS can serve as a valuable tool for the analysis of cellulose microfibril orientation in primary cell walls and, in consequence, add to the understanding of its mechanical behaviour and the intriguing mechanisms behind cell growth.

## Background

Cellulose microfibril orientation plays an important role for plant cell wall mechanics and determines cell shape during growth. A number of complementary techniques can be used to show the arrangement of cellulose fibrils: transmission electron microscopy provides details with very high resolution; the field of view, however, is small [1,2]. In field emission scanning electron microscopy and atomic force microscopy, only the newly synthesised microfibrils at the inner face of the cell walls are accessible [3-6]. Polarised Fourier transform infrared microscopy yields one predominant microfibril orientation and can be extended to show a distribution of microfibril orientations [7]. Nevertheless, the method is not well suited for the application on primary cell walls as little wall material is available and oblique cellulose microfibril orientations can further impede the experiment. Fluorescent cellulose dyes like Pontamine Fast Scarlet 4B can be used to visualize the cellulose architecture in cell walls by confocal microscopy. While the dyes easily penetrate into root cells, it remains a challenge to apply the technique to intact hypocotyls [8]. Chemical and genetic analyses can shed light on composition and assembly [9,10], but a method to analyze the bulk of microfibril orientation of intact primary plant cell wall tissues is lacking.

SAXS allows the characterization of structures within the size range of 0.5 nm to 100 nm; this also applies to cellulose microfibrils. Only minor sample preparations are required and high statistical accuracy results from averaging over a large sample volume. Scanning the sample with a microbeam enables spatially resolved measurements of the local microfibril orientation.

**Figure 1:**
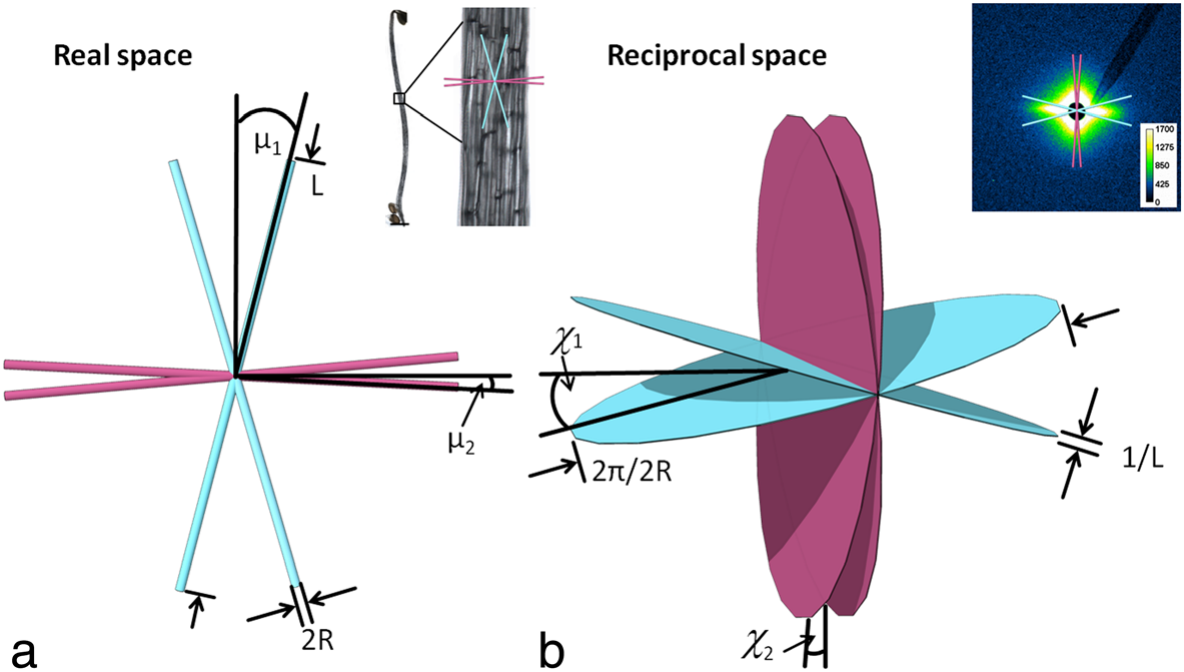
**Principle of small angle X-ray scattering of cellulose microfibrils. a)** Cellulose microfibrils in real space. Orientations are shown on the sample image at the upper right. Scale bar: 2 mm **b)** Fourier transform of the microfibrils are flat disks in reciprocal space that appear as two-dimensional scattering pattern on the detector (upper right). R: microfibril radius, L: microfibril length, μ: microfibril angle, α: cell wall orientation, χ: azimuthal peak positions in reciprocal space. Scalebar 2 mm. For simplicity only the calculated mean orientations are shown. Drawing adapted from [13].

The SAXS-signal in plant tissues originates from differences in electron densities between the cellulose microfibrils and the surrounding matrix. It appears in close proximity of the primary beam at q < 5 nm^-1^, q being the modulus of the scattering vector $\vec{q}$, which is the difference between the vector of the incoming and the scattered X-ray beam. For wet samples, the SAXS signal is dominated by the scattering of the cellulose fibrils; contributions of pores can be neglected [11,12]. In SAXS, the long and rod shaped cellulose fibrils appear as flat disks in the scattering pattern, corresponding to the Fourier transform in reciprocal space. Hence, the scattering image emanating from a collection of fibrils in the cell wall represents the superposition of all disks originating from the different cellulose fibrils within the irradiated sample volume. The image appearing on a two-dimensional detector is a cut through the three-dimensional figure corresponding to these superposed disks (illustrated in Figure 1, [13]). The azimuthal intensity distribution of the resulting streaks is used to extract information on the distribution of microfibril orientation [14-16].

Measurement of cellulose microfibril orientation through small angle X-ray scattering (SAXS) is well established for the comparably thick secondary cell walls in which cellulose fibrils are mainly oriented in parallels [14,17,18]. This architecture leads to a strongly anisotropic SAXS signal from which mean orientations of the cellulose fibrils in secondary cell walls can be calculated [14].

For primary cell walls, Bayley et al. [19] obtained oriented SAXS patterns from the thick cell walls of *Avena* coleoptiles and deduced preferential orientations. Hydration dependency of microfibril spacing was investigated by SAXS on primary cell walls of celery collenchyma [20]. However, the primary cell walls of the model organism *Arabidopsis thaliana* are much thinner [21], resulting in weak scattering signals. To our knowledge, only one X-ray scattering study on the structure of secondary cell walls of *Arabidopsis*[22] exists. To date, no X-ray data on structure and orientation distribution of microfibrils in primary cell walls of *Arabidopsis* are available.

**Figure 2:**
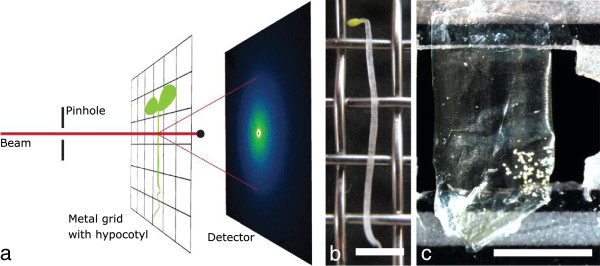
**Experimental setup for small angle X-ray scattering. a)** Setup at the μ-spot beamline (BESSY II). Beam size is adjusted by the pinhole before it hits the sample. The scattered signal is collected on a 2D-detector while the primary beam is kept back by a beam-stop. **b)** Sample preparation: 6d old, dark grown *Arabidopsis thaliana* hypocotyls are mounted onto a metal grid in their hydrated state. **c)***Chara corallina* cell is cut open and a single cell wall is placed in the beam. White spots at the lower right of the sample are calciumcarbonate depositions (not relevant for the microfibril measurement). Scalebars: 2 mm.

In this paper, we present SAXS analysis of primary cell walls from *Chara corallina* and *Arabidopsis thaliana.* The large cells of *Chara* enable the analysis of a single primary cell wall whereas for *Arabidopsis* data are obtained from the complex, multicellular hypocotyl. We deduce a microfibril angle distribution for both plants. The measurements were performed at the μ-spot beam line of the BESSY II synchrotron radiation facility (see Figure 2 for the setup). To obtain the microfibril angle distribution we adapted the simulation procedure of Rueggeberg et al. [23] which was developed for WAXD experiments on the basis of the simulation of Entwistle et al. [24]. Cell geometry, peak broadening and cellulose microfibril orientation distributions (not exclusively Gaussians) can be taken into account [23]. In addition, an analytical solution assuming cylindrical cell geometry was implemented to verify the results [15,16].

## Results

**Figure 3:**
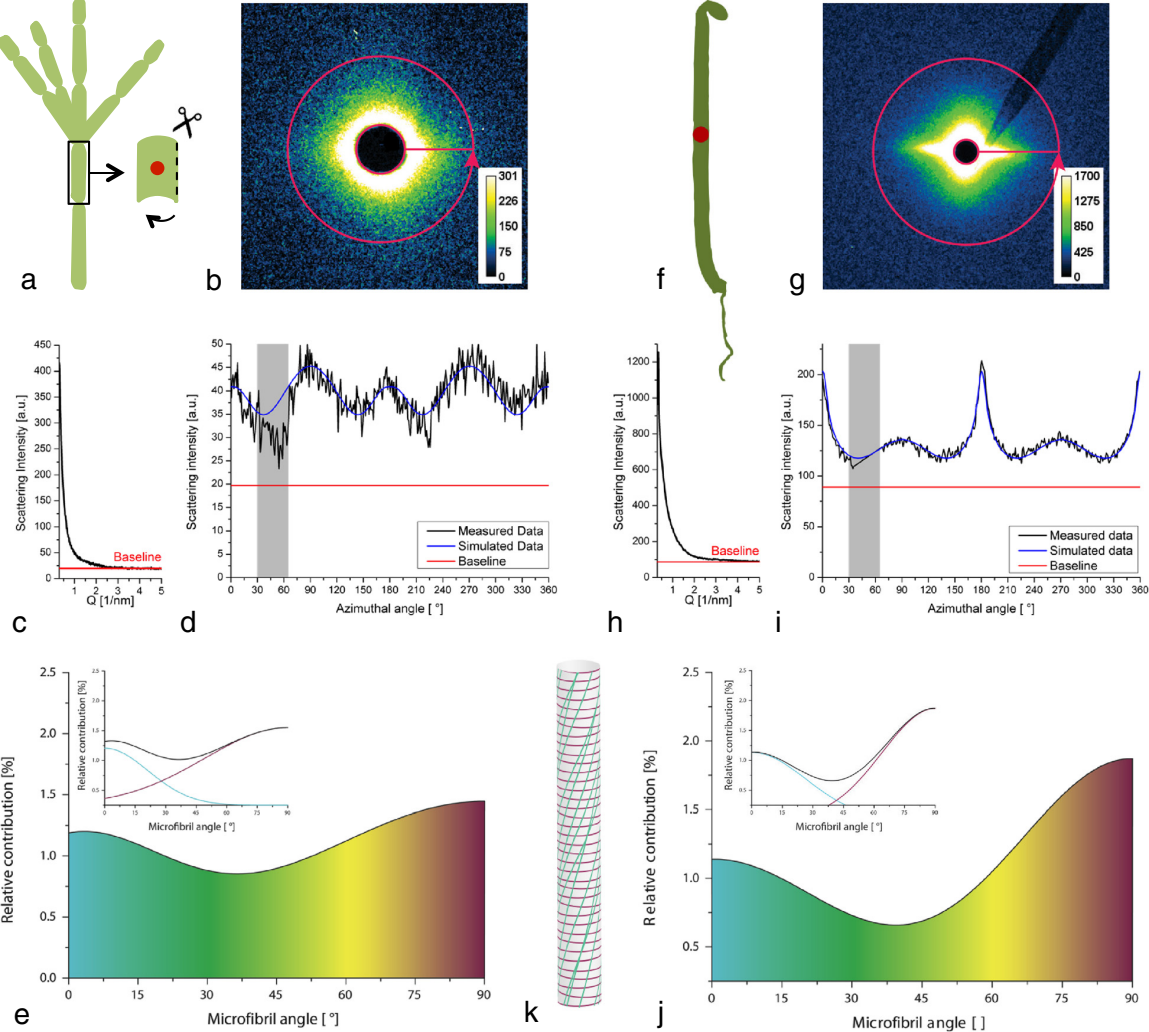
**SAXS analysis.a-e: *Chara corallina*. a)** One cell was cut open and a single cell wall was analysed. **f-j**: Primary cell walls of *Arabidopsis thaliana*. **a** and **f**) 6 day old dark grown hypocotyl is analysed. The beam diameter is close to the hypocotyl diameter. **b** and **g**) 2D scattering image: 360° azimuthal integration is marked. The scattering signal originates from electron density contrast between the cellulose microfibrils and water. **c** and **h**) The radial scattering profile is used to assign the background of the measurement. **d** and **i**) Integrated scattering signal (measured data) can be fitted by microfibril angle simulation (simulated data). The area beneath the baseline as well as the data range influenced by the glass capillary on which the beamstop was mounted (grey area) are not considered in the simulation. **e** and **j**) Relative contribution of each microfibril angle to the simulated scattering signal. The colour gradient represents the microfibril orientations from 0° (longitudinal towards the cell axis, indicated in blue) to 90° (transverse to the longitudinal axis of the cell, indicated in red). The insets show the two underlying distributions. At intermediate microfibril angles there is an overlap in which the two distributions cannot be distinguished. **k)** Schematic of a possible arrangement of the microfibrils in a cell with the colour scheme used in **e** and **j**.

Typical SAXS scattering patterns and the corresponding radial and azimuthal intensity distributions are shown in Figure 3 for *Chara corallina* and *Arabidopsis thaliana*. The contribution of background scattering from amorphous cellulose and other cell wall components can be accounted for by setting a baseline at the minimum scattering intensity at a q-value of approximately 4 nm^-1^ in the radial profile (Figure 3c and h). In the azimuthal intensity distribution (Figure 3b and g), which is used for orientation analysis, the area underneath the corresponding baseline (marked by the red line in Figure 3d and i) is not considered in the simulation. A theoretical intensity profile is calculated based on cell geometry and then fitted to the measured intensity distribution by varying the contribution of one or several cellulose orientation distributions according to Rueggeberg et al. [23]. In the present case, two Gaussian distributions were necessary to obtain appropriate fits.

A single unfolded cell wall of *Chara corallina* was analysed (Figure 3a). Two preferential orientations at ~0° and ~90° with respect to the longitudinal axis of the cell were found (Figure 3e and k). The peak areas correspond to the relative contribution of the microfibril angles to the total scattering signal. The distribution with its mean value at 90° (transversely oriented microfibrils) covered about 72% of the total area. Only about 28% of the scattering signal originated form the second microfibril distribution with a mean angle of around 0° with respect to the longitudinal axis of the cell. At intermediate microfibril angles the distributions overlap. This is indicated by the colour gradient in Figure 3e and j.

For *Arabidopsis thaliana* a section in the lower middle region of the hypocotyl was chosen for analysis (Figure 3f-i). As the beam diameter was approximately half the diameter of the hypocotyl, the results of the measurements represent an average of all the cells in the irradiated section. About 38% of the microfibrils were oriented with a mean angle of approximately 0° towards the cells axis while the remaining 62% were oriented transversely with a mean angle of approximately 90° towards the cells axis (Figure 3j and k).

For both organisms, the distributions of the cellulose microfibrils were bimodal and fairly broad. Contributions were detected from all angles within a range of 0 - 90° with respect to the longitudinal axis of the cell wall.

**Figure 4:**
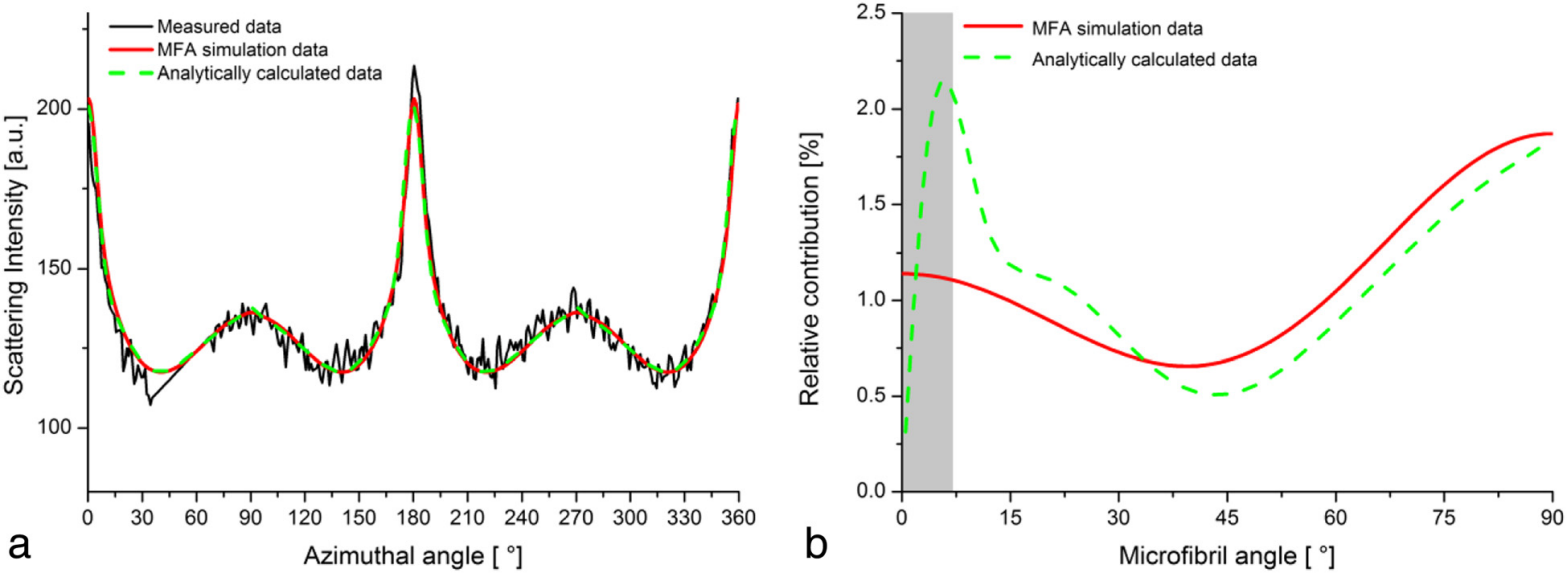
**Comparison of the methodologies for *Arabidopsis thaliana *hypocotyl. a)** Measured and calculated data for the simulation procedure and the analytically calculated solution. Measured data and fits are in good accordance for both methods. **b)** The resulting microfibril angle distributions. Deviations can be found especially at small microfibril angles where the real microfibril angle distribution can be approximated with less accuracy.

The orientation analysis through the simulation procedure under the assumption of cylindrical cells was compared to the calculated analytical solution for *Arabidopsis thaliana*. Both methods yielded a good quality of the fits (Figure 4a). At small angles however, the two distributions show differences in the microfibril angle distribution (Figure 4b). To further investigate the differing results at small microfibril angles, two theoretical scattering patterns were analysed. For equal scattering intensities, the obtained microfibril angle distributions for both methods, simulation procedure and analytical solution, corresponded well (Additional file 1: Figure S1a and b). For the theoretical intensity profile of a uniform distribution of microfibrils, the results of both methods deviate strongly from the theoretical, uniform distribution at small microfibril angles (Additional file 1: Figure S1c and d).

## Discussion

SAXS provides the possibility to obtain data on the orientation distributions of cellulose microfibrils on native plant material. Depending on the chosen size of the X-ray beam, it is possible to either scan the sample with a spatial resolution down to the μm-range or to obtain an average over a larger sample volume containing many cells. However, the benefit of averaging over several cells requires some knowledge of the system at hand, as the signal represents an overlay of possibly different distributions of microfibril angles. The method cannot distinguish between two cell types with different distributions and one cell type showing two distributions. Therefore, alterations in cellulose microfibril orientation in the cell walls of different cell types or in different cell wall layers cannot be resolved. In multi-scale systems consisting of different cell types like the *Arabidopsis* hypocotyl the origin of the scattering signal can only be assigned by additional measurements e.g. after separation of different tissue types or by microscopy. The scattering image is not only averaged spatially, but also temporally, through the measurement time. Due to radiation damage it thus remains difficult to capture highly dynamic processes.Scattering intensities of the presented model systems are low compared to intensities obtained from wood sections due to the small amount of material in primary cell walls. However, the fitting procedure used to calculate cellulose orientation distributions tolerates a low signal to noise ratio (Figure 3d). Using synchrotron radiation has the advantage of a high brilliance of the beam and thus shorter measurement times. In principle though, the experiments can also be performed on a laboratory instrument.

In SAXS measurements, the scattering contrast is linked to the electron density difference between cell wall components. Hence, samples should be kept in the hydrated, native state. The reason being that the method is only applicable when the scattering of the microfibrils dominates the signal and additional contributions (e.g. arising from cracks or pores) are suppressed. In the case of hydrated plant cell walls, the highest electron density contrast exists between crystalline cellulose fibrils and the surrounding water-saturated matrix. Thus, a two-phase system is a good approximation and can be used as a basis for further analysis [11,12]. Upon drying, air-filled voids or cracks might appear and blur the scattered signal due to their strong electron density difference with respect to hydrated matrix.

For this study, two methods of data analysis were compared. The analytical solution is valid under the assumption of cylindrical cells, while the simulation procedure offers the advantage that other cell shapes can be considered as well. In the simulation procedure, the microfibril angle distribution is approximated by two Gaussian peaks to limit the number of fit parameters to the necessary minimum. When using the analytical solution, the number of degrees of freedoms is generally lower and more details on the microfibril angle distribution can be included into the fitting procedure. To increase the quality of the fit in the simulation procedure, it is possible to increase the number of Gaussian peaks approximating the microfibril angle distribution. Yet, a balance between the detailed representation of features of the measured data by the fit and the increase in fitting parameters has to be found. Both methods become imprecise in the range of very small microfibril angles. The analytical calculations of the microfibril angle distributions are valid for spherical cells and the results are then transferred to a cylindrical geometry. As the radius of a sphere in the direction normal to the vertical axis becomes infinitely small at the poles, the intensity of the scattering originating from the microfibrils in this region should in theory be concentrated in a point and thus become very high. Thus, in the case of small microfibril angles, the scattering pattern contains narrow peaks. However, due to instrumental broadening of the beam, these features are less pronounced in the measured data and depending on the chosen setup, the first degrees of the microfibril angle distribution – in this case , it is the first 5-7° [25] - cannot be accurately determined.

The effect of large scattering contributions of small microfibril angles is strongest for an equal distribution of all microfibril angles to the total scattering signal. The deviation of the simulated and analytically calculated solutions from the theoretical solution gives the range within which the results can maximally vary. Decreasing the interval of the azimuthal angle used for calculating the intensity distribution in the analytical solution further enhances the deviation from the theoretical result. The reason for this being that the theoretical scattering pattern approaches infinite values for microfibril angles close to zero, which cannot accurately be represented by the cosine functions used to fit the data (Equation 4).

## Conclusions

The methods described here, allow for analysis of the cellulose microfibril orientation distributions within primary cell wall systems in their native state. The primary cell walls of *Arabidopsis thaliana* and *Chara corallina* both show a bimodal microfibril angle distribution such that the bulk of the microfibrils is oriented either transversely or longitudinally with a broad scattering. However, the scattering signal does not contain information on the orientation of microfibrils at small microfibril angles (up to 7° towards the longitudinal axis of the cell), which has to be taken into consideration when applying the method. The SAXS data can supplement the existing information on cell wall structure at different growth stages and thus, help to understand the complex mechanisms behind cell wall formation and cell growth.

## Methods

### Material

*Chara corallina* Klein ex Willd., em. R.D.W.( = C. australis R. Br.) was grown in the laboratory in 120 dm^3^containers in a solution of distilled water with 1.0 mM NaCl, 0.2 mM KCl, 0.2 mM CaSO_4_x2H_2_O and 1.0 mM NaHCO_3_titrated to pH 8.2 by NaOH according to [26].

*Arabidopsis thaliana* (L.) hypocotyls of the ecotype Col-0 were investigated in this study. The sterilized seeds were plated in 2 g/l Murashige and Skoog basal medium in agar and incubated in the dark at 22 °C for 6 days.

### X-ray scattering

X-ray measurements were performed at the μ-spot beamline at the synchrotron radiation facility of the Helmholtz-Zentrum Berlin für Materialien und Energie (BESSY II). The setup consists of a beam-defining pinhole, a guard pinhole and a two-dimensional position-sensitive CCD-detector MarMosaic 225 (Mar USA Evanston, USA) [25]. Fresh, hydrated *Arabidopsis* hypocotyls were mounted onto a metal grid (mesh size 2.5 mm; Figure 2b) through capillary forces and placed in the beam (transmission mode) (Figure 2a). From *Chara corallina* a 3 cm long cell was chosen for analysis, cut open on one side, unfolded and taped to a foliar frame in the hydrated state (Figure 2c). During the measurement the samples were kept wet by a humidifier. To achieve an integration over several cell walls the X-ray beam diameter was adjusted to ~100 μm, which is about half of the average diameter of an *Arabidopsis* hypocotyl. The wavelength was set to 0.82656 Ångstrom (energy of 15 keV) and typical measuring times were 60 s. The sample-detector distance was set to 320 mm for *Arabidopsis* (simultaneous detection of the SAXS and WAXD signal) and to 840 mm for Chara (detection of the SAXS signal only) and the q-axis was calibrated against a Quartz (WAXD) or Silverbehenate (SAXS) standard. A background reference frame taken without sample allowed for subtracting air scattering. The signals were also corrected for the instrumental noise (dark current).

In principal, if wide angle X-ray diffraction (WAXD) is recorded simultaneously with SAXS it may also be used for analyzing cellulose orientation. However, the WAXD signal of primary cell walls is much weaker compared to the SAXS signal due to low cellulose crystallinity in primary walls. The SAXS signal contains fewer disturbances from other components like cuticular waxes and in samples analyzed in the hydrated state, the main WAXD signal of cellulose is masked by the water halo. Thus, the sample can only be analyzed in the dry state, where the drying process might have induced changes in the fragile cell wall.

### Data analysis

Beam centre, detector tilt and the sample-detector distance were determined using the Quartz and Silverbehenate measurements by the calibrant routine implemented in Fit2D [27]. Integration of the sample data was automated using Fit2D and Autofit, a Python based software developed by Chenghao Li, Aurelien Gourrier and Gerald A. Zickler at the Max-Planck-Institute of Colloids and Interfaces in Potsdam. The SAXS signal was integrated in two ways: Azimuthal integration gives the scattering intensity as a function of the scattering vector q (Figure 3b and g) and can be used to obtain structural information. To obtain information on microfibril orientation, the scattering signal is integrated radially, which results in the intensity as a function of the azimuthal angle χ. The intensity of the scattering signal as a function of the scattering vector q is high in the q-region of 0.3 – 2 nm^-1^ and then drops quickly to run into a minimum at values of q around 3 nm^-1^. This minimum was used to assign the baseline for the following simulation. The SAXS q-region is then integrated azimuthally from 0-360° as shown in Figure 3c and g. Oriented cellulose microfibrils, amorphous non-oriented cell wall components as well as the background contribute to the azimuthal signal.

#### ***Simulation procedure***

The microfibril angle distribution could be calculated from the azimuthal signal similar to the procedure described for wide angle X-ray diffraction (WAXD) in Rüggeberg et al. [23]. However, the equation for calculating the position χ of the intensity maxima on the detector in dependency of μ and α differs from that for WAXD. It has been deduced by several authors [18,28] and is given in Equation 1.

(1) tanχ=tanμ·cosα

α is the cell wall orientation and μ the microfibril angle with respect to the longitudinal axis of the cell (Figure 1). For a single cell wall, like in the case of *Chara* that is placed perpendicular to the beam, the cell wall orientation α is 0 and the microfibril angle can be deduced directly from the scattering image as χ= μ. For *Arabidopsis* cylindrical cells are assumed and the peak positions are calculated for every combination of μ and α in 1° steps. Fit parameter is the contribution of every microfibril angle μ to the scattering signal with the restriction that the resulting microfibril angle distribution can be described by two Gauss peaks.

#### ***Analytical solution***

For cylindrical cells it is possible to obtain an analytical solution. The variation of spiral angles within a cylindrical cell, in which the microfibril angle μ varies between –π/2 and π/2, is called g(μ). The microfibril angle distribution on a cylinder h(μ) is because of the symmetry of the round cell determined as h(μ) = g(μ) + g(-μ) with μ varying between 0 and π/2. The function is normalized such that $\;\int_{0}^{\frac{\pi }{2}}h\left(µ\right)dµ\;=\;1.$ The probability p(μ) that a cellulose fibril has an angle μ on a sphere is related to the microfibril angle h(μ) on a cylindrical cell by h(μ) = p(μ)sinμ. To deduce the microfibril angle distribution the equation for the intensity distribution given by [16] is inverted:

(2) $$I\left(\chi \right)=\frac{2}{\pi }\int_{\chi }^{\frac{\pi }{2}}\frac{p\left(µ\right)\text{sin}\left(µ\right)}{\sqrt{\mathrm{co}s^{2}\chi -\mathrm{co}s^{2}µ}}dµ=\frac{2}{\pi }\int_{\chi }^{\frac{\pi }{2}}\frac{h\left(µ\right)}{\sqrt{\mathrm{co}s^{2}\chi -\mathrm{co}s^{2}µ}}dµ.$$

The inversion of the function can be done numerically [16], yet since I(χ) can only be determined from the detector image with limited precision, the SAXS data can be fitted by:

(3) $$I\left(\chi \right)=\sum_{\left(n=1\right)}^{m}a_{n}{\text{cos}}^{2\gamma_{n}}\chi \mathrm{for}\left(0\leq \chi \leq \frac{\pi }{2}\right)\mathrm{and}\gamma_{n}\geq 0.$$

Then the microfibril angle distribution p(μ) becomes:

(4) $$p\left(µ\right)=\sum_{n=1}^{m}b_{n}{\text{cos}}^{2\gamma_{n}}µ\text{with}b_{n}=\frac{2a_{n}\Gamma \left(\gamma_{n}+1\right)/\Gamma \left(\gamma_{n}+\frac{1}{2}\right)}{\sum_{k\geq 0}a_{k}\Gamma \left(\gamma_{k}+1\right)/\Gamma \left(\gamma_{k}+\frac{3}{2}\right)}$$

(see appendix of [15]).

The measured data is fitted using Equation 3 and due to the symmetry of the scattering signal, averaging the four sectors is possible. In the next step the microfibril angle distribution of a cylindrical cell h(μ) can be calculated from p(μ). The intensity distribution can be calculated for arbitrary intervals of the azimuthal angle. In this case intervals of 1° were chosen which is a reasonable compromise between precision and calculation time.

The simulation procedure has been written in Excel and Python and is available from the authors on request.

## Competing interests

The authors declare that they have no competing interests.

## Authors’ contributions

FS, GB and MR carried out the X-ray measurements, data analysis and drafted the manuscript. FS, ME, GB, BA, IB, PF and MR discussed analyses, interpretation, and presentation. FS, MR and IB designed and coordinated the study. All authors read and approved the final manuscript.

## Supplementary Material

Additional file 1: Figure S1 Analysis of calculated theoretical scattering profiles. **a)** Fit of analytical solution and simulation procedure for equal scattering intensities over the entire azimuthal profile. **b)** The resulting microfibril angle distributions from both methods are in very good accordance. **c)** Fit of analytical solution and simulation procedure for the theoretically calculated scattering intensities of round cells with an equal distribution of all microfibril angles. Inlet: The resulting azimuthal scattering profile (green) of the simulation procedure has two contributing distributions (light and dark blue curves) as two Gauss peaks were used to fit the microfibril angle distribution. **d)** The resulting microfibril angle distributions from both methods deviate from the theoretical, uniform distribution. For microfibril angles larger than 7°, minor deviations can be observed that are a measure for the achievable accuracy of the results. The grey areas indicate the region of small microfibril angles in which the orientation cannot accurately be determined due to the instrumental broadening of the incident beam. Click here for file
