# Supplementary figures and images for: Measuring the distribution of cellulose microfibril angles in primary cell walls by small angle X-ray scattering
Source: Plant Methods. 2014 Aug 5;10:25. doi: 10.1186/1746-4811-10-25 (PMC4137751; doi:10.1186/1746-4811-10-25)

## Slide 1
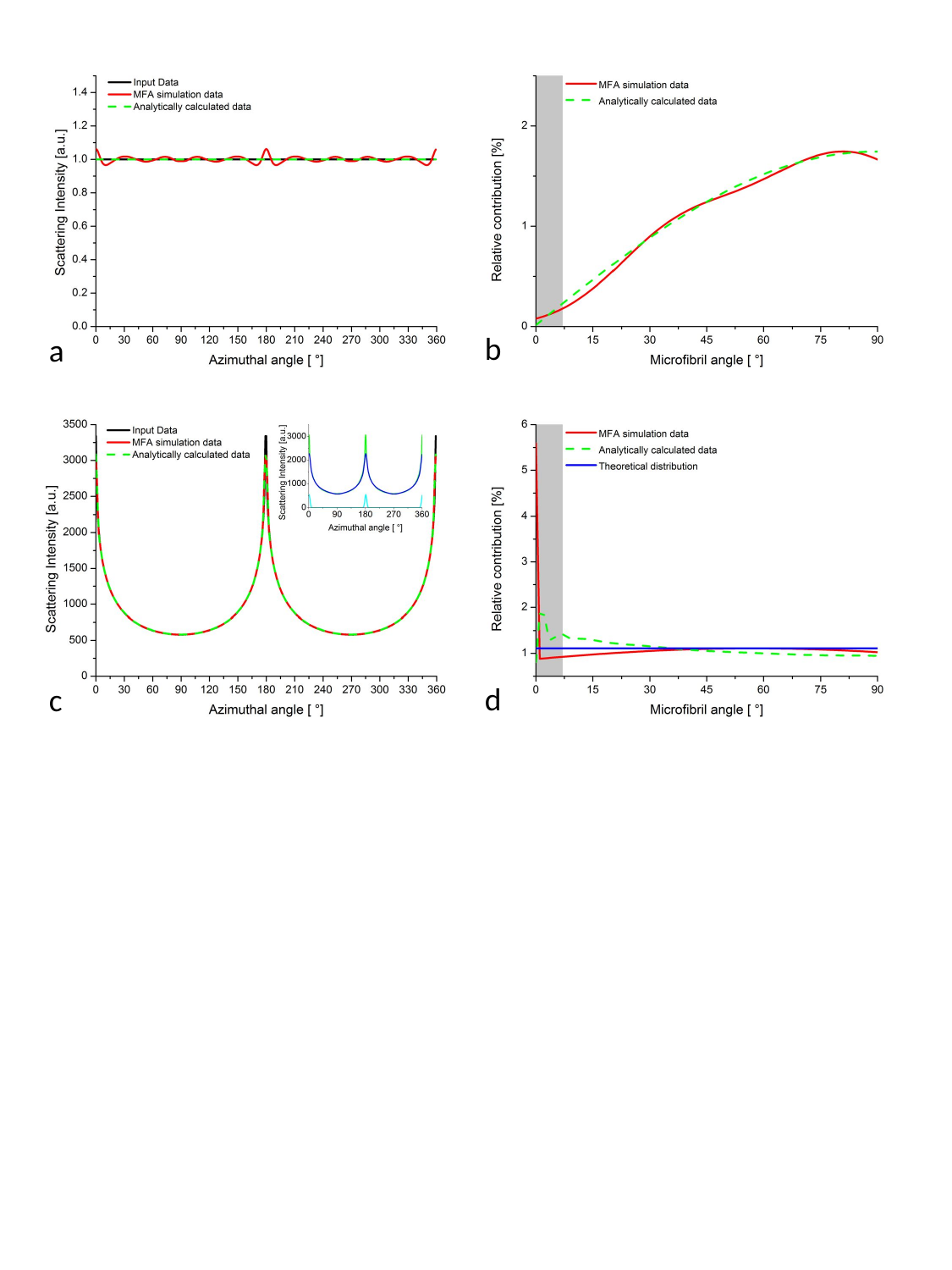

b
a
d
c

Supplement: Additional file 1: Figure S1 — Analysis of calculated theoretical scattering profiles. a) Fit of analytical solution and simulation procedure for equal scattering intensities over the entire azimuthal profile. b) The resulting microfibril angle distributions from both methods are in very good accordance. c) Fit of analytical solution and simulation procedure for the theoretically calculated scattering intensities of round cells with an equal distribution of all microfibril angles. Inlet: The resulting azimuthal scattering profile (green) of the simulation procedure has two contributing distributions (light and dark blue curves) as two Gauss peaks were used to fit the microfibril angle distribution. d) The resulting microfibril angle distributions from both methods deviate from the theoretical, uniform distribution. For microfibril angles larger than 7°, minor deviations can be observed that are a measure for the achievable accuracy of the results. The grey areas indicate the region of small microfibril angles in which the orientation cannot accurately be determined due to the instrumental broadening of the incident beam. [file 1746-4811-10-25-S1.pptx]
